# Survival after Abdominoperineal and Sphincter-Preserving Resection in Nonmetastatic Rectal Cancer: A Population-Based Time-Trend and Propensity Score-Matched SEER Analysis

**DOI:** 10.1155/2017/6058907

**Published:** 2017-01-18

**Authors:** Rene Warschkow, Sabrina M. Ebinger, Walter Brunner, Bruno M. Schmied, Lukas Marti

**Affiliations:** ^1^Department of Surgery, Cantonal Hospital of St. Gallen, 9007 St. Gallen, Switzerland; ^2^Institute of Medical Biometry and Informatics, University of Heidelberg, 69120 Heidelberg, Germany; ^3^Department of Surgery, Hospital of Thun, 3600 Thun, Switzerland; ^4^Department of Surgery, Universitätsmedizin Mannheim, Medical Faculty Mannheim, University of Heidelberg, 68167 Mannheim, Germany

## Abstract

*Background.* Abdominoperineal resection (APR) has been associated with impaired survival in nonmetastatic rectal cancer patients. It is unclear whether this adverse outcome is due to the surgical procedure itself or is a consequence of tumor-related characteristics.* Study Design.* Patients were identified from the Surveillance, Epidemiology, and End Results database. The impact of APR compared to coloanal anastomosis (CAA) on survival was assessed by Cox regression and propensity-score matching.* Results.* In 36,488 patients with rectal cancer resection, the APR rate declined from 31.8% in 1998 to 19.2% in 2011, with a significant trend change in 2004 at 21.6% (*P* < 0.001). To minimize a potential time-trend bias, survival analysis was limited to patients diagnosed after 2004. APR was associated with an increased risk of cancer-specific mortality after unadjusted analysis (HR = 1.61, 95% CI: 1.28–2.03, *P* < 0.01) and multivariable adjustment (HR = 1.39, 95% CI: 1.10–1.76, *P* < 0.01). After optimal adjustment of highly biased patient characteristics by propensity-score matching, APR was not identified as a risk factor for cancer-specific mortality (HR = 0.85, 95% CI: 0.56–1.29, *P* = 0.456).* Conclusions.* The current propensity score-adjusted analysis provides evidence that worse oncological outcomes in patients undergoing APR compared to CAA are caused by different patient characteristics and not by the surgical procedure itself.

## 1. Introduction

Abdominoperineal resection (APR) has long been considered the standard of care for curative treatment of distal rectal cancer. Recently, this dogma has been increasingly questioned [1–3]. Besides the fact that APR defines the sphincter's fortune by creating a permanent colostomy, it has also been associated with an impaired oncological outcome and survival compared to restorative operations [4–6], even if performed for distal rectal cancer with coloanal anastomosis (CAA) [7].

Decision-making for sphincter preservation versus sphincter resection is related to numerous tumor- and patient-related characteristics. Whether an adverse outcome is due to one or a combination of these factors or to the surgical procedure of APR itself is a matter of debate [8]. A prominent factor is a tumor's distance to the anal sphincters. Because a wide distal margin has formerly been considered to be of particular importance, tumors less than 5 cm from the anal verge could not be operated on except by APR. Because local recurrence and overall survival were then proved not to be impaired by a limited margin, the recommended distal resection margin was incrementally reduced from 5 cm to 1 cm [9–11] and even to 0.5 cm in special cases of tumors that were downstaged after neoadjuvant chemoradiotherapy [12]. However, in patients with higher tumor grades, a broader distal margin is recommended [13]. Other factors that might lead to performing an APR and not a sphincter-preserving procedure are higher T-stage [14, 15], male gender with a narrow pelvis [14, 16], higher age [17], and impaired preoperative sphincter function to avoid postoperative incontinence [16].

In the literature, there are contradictions about the impact of APR on oncological outcome and survival [1, 6, 17–19]. Of note, some of the factors favoring APR over restorative operations (e.g., T-stage, age, and distance to the anal verge) are independent risk factors for poor oncologic outcome after APR [5]. Additionally, the rate of APR has decreased significantly during the last two decades [20]. Hence, a comparison of APR versus CAA should consider such a selection and time-trend bias.

Therefore, the aims of the current population-based investigation were to first define the optimal study period by time-trend analysis and then to assess the putative impact of APR versus CAA on survival in unadjusted and multivariable Cox proportional hazard regression analyses. Finally, a statistically optimal adjustment for imbalances in patient characteristics was undertaken by propensity score matching to further elaborate the prognostic impact of APR.

## 2. Materials and Methods

### 2.1. Cohort Definition: Surveillance, Epidemiology, and End Results

Data from the Surveillance, Epidemiology, and End Results (SEER) Program of the National Cancer Institute in the United States, covering approximately 28% of cancer cases in the United States, were the source of the present population-based analysis [21]. The SEER data were collected and reported using data items and codes as documented by the North American Association of Central Cancer Registries (NAACCR) [22]. Primary cancer site and histology were coded according to the criteria in the third edition of the International Classification of Diseases for Oncology (ICD-O-3) [23]. Rectal cancer patients were identified by the ICD-O-3 site code C20.9 and behavior code 3 (NAACCR Items 522 and 523). Patients diagnosed at autopsy or only by death certificate were excluded, as well as patients without histologically confirmed cancer (NAACCR Items 490 and 2180) and patients with occurrence of another malignancy preceding rectal cancer (NAACCR Item 380). The analysis was further restricted to patients with adenocarcinoma identified by the ICD-O-3 histology codes 8140, 8144, 8210, 8211, 8220, 8221, 8261, 8262, and 8263 (NAACCR Item 522), patients without distant metastases (NAACCR Item 790 in 1998 to 2003 and Item 3000 in 2004 to 2011), and patients without intraoperative radiation (NAACCR Item 1360). For trend analysis, patients with any rectal cancer resection were included (NAACCR Item 1290, codes 30 to 80). To analyze the impact of APR on prognosis, the cohort was further limited to patients diagnosed between 2005 and 2011 and undergoing either APR or rectal cancer resection with sphincter preservation and CAA (NAACCR Item 1290, codes 50 and 40). Patients undergoing rectal cancer resection with colorectal anastomosis were not included in the survival analysis because they were mixed with patients undergoing anterior resection without complete mesorectal excision and patients treated with Hartmann's procedure (NAACCR Item 1290, code 30).

### 2.2. Statistical Analysis

Statistical analyses were performed using R statistical software (https://www.r-project.org/). A two-sided *P* value < 0.05 was considered statistically significant. Continuous data are expressed as medians (interquartile range). Chi-square statistics and Mann–Whitney *U* tests were used to compare proportions and continuous variables. In regression analysis, all *P* values were computed by likelihood-ratio tests. Wald-type confidence intervals were estimated.

To analyze the time trend in the APR rate, logistic regression and Davis tests [24] were applied to test for points in time at which a significant change in APR rate had occurred. Joinpoint regression analysis [25] was applied to define the best fitting point for a change in the time trend of the APR rate. The trends in the two segments defined by the joinpoint were characterized by the annual percentage change [25]. For sensitivity analysis, the time trend was finally assessed by LOESS regression analysis [26].

After comparing patients with APR and CAA in descriptive analysis, APR was assessed as a prognostic factor for overall and cancer-specific survival in Kaplan-Meier analysis and in Cox regression analyses with and without risk adjustment for tumor stage according to the American Joint Committee on Cancer (AJCC, 6th edition) for retrieved regional lymph nodes, grading, year of diagnosis, age, gender, ethnicity, and marital status (risk set). The full model Cox regression was further elucidated by a backward variable selection procedure from the full model based on Akaike's information criterion. The proportional hazard assumption was tested by scaled Schoenfeld residuals and by inspection of the hazard ratio (HR) plots [27]. Thereafter, predictors of APR in the risk set were assessed in multivariable logistic regression to assess the bias concerning APR. Moreover, a propensity score analysis was performed as a superior and more refined statistical method to adjust for all potential baseline-confounding variables in the risk set [28–30]. Propensity score matching was performed as exact matching. In this procedure, each patient undergoing APR was matched to all possible patients undergoing CAA with exactly the same values on all the covariates, forming subclasses such that within each subclass both groups had exactly the same covariate values after assigning weights to each individual. Patients undergoing APR who did not have a counterpart among the patients undergoing CAA and vice versa were excluded from this analysis. Finally, overall and cancer-specific survival in patients undergoing APR was assessed in a Cox regression analysis using the weights obtained by the matching propensity score analysis.

## 3. Results

### 3.1. Trend Analysis

**Figure 1:**
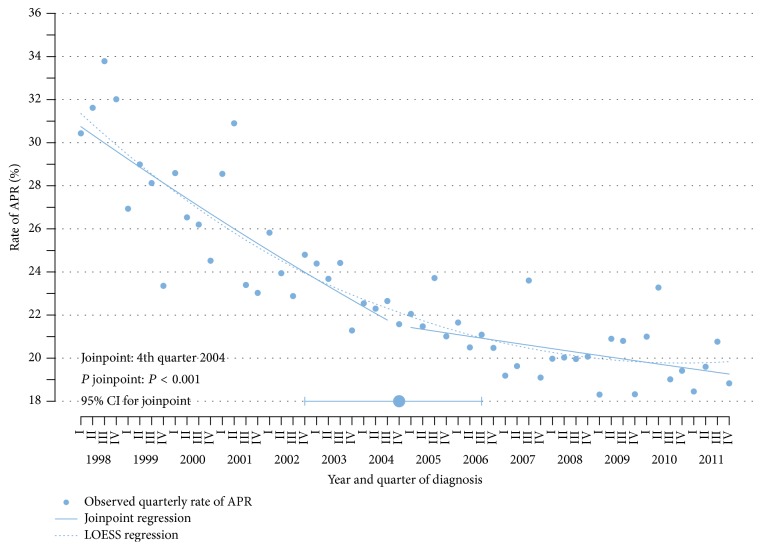
Trend analysis for abdominoperineal resection, 1998 to 2011.

The trend analysis was based on 36,488 patients who underwent resection of nonmetastatic rectal adenocarcinoma. The rate of APR declined significantly from 31.8% in 1998 to 19.2% in 2011 (*P* < 0.001). Further analyzing this trend (Figure 1), joinpoint regression analysis identified one notable change in the APR rate at the 4th quarter of 2004 (*P* < 0.001). The 95% confidence interval for this break in the time trend was estimated to be between the 4th quarter of 2002 and the 3rd quarter of 2006. There was no evidence for additional relevant changes in the trend (*P* = 0.716). From the 1st quarter of 1998 until the 4th quarter of 2004, the observed rate of APR declined from 30.4% to 21.6%, corresponding to an annual percent change of −7.1% (95% CI: −9.1% to −5.2%, *P* < 0.001). Thereafter, the rate of APR declined further to 18.8% at the 4th quarter of 2011 (*P* = 0.018), but to a much lower extent. The annual percent change after 2004 was −2.0% (95% CI: −3.6% to −0.3%). A LOESS regression was performed for sensitivity analysis and confirmed a lower decline after 2004 (Figure 1).

### 3.2. Patient Characteristics for Abdominoperineal Resection

The comparative analysis of oncologic outcomes after APR versus CAA was limited to patients diagnosed after 2004 to minimize a potential time-trend bias, leaving 4,700 patients eligible for this part of the analysis. Of these, 3,898 patients (82.9%) underwent APR and 802 (17.1%) underwent rectal resection with CAA.  Table 1 summarizes the patient characteristics for both groups. Patients with APR had more advanced cancer stages, less regional lymph nodes retrieved, more advanced grading, and more applications of radiotherapy, were significantly older, were less often African-Americans, and were less often married.

### 3.3. Abdominoperineal Resection as a Prognostic Factor for Survival

**Figure 2:**
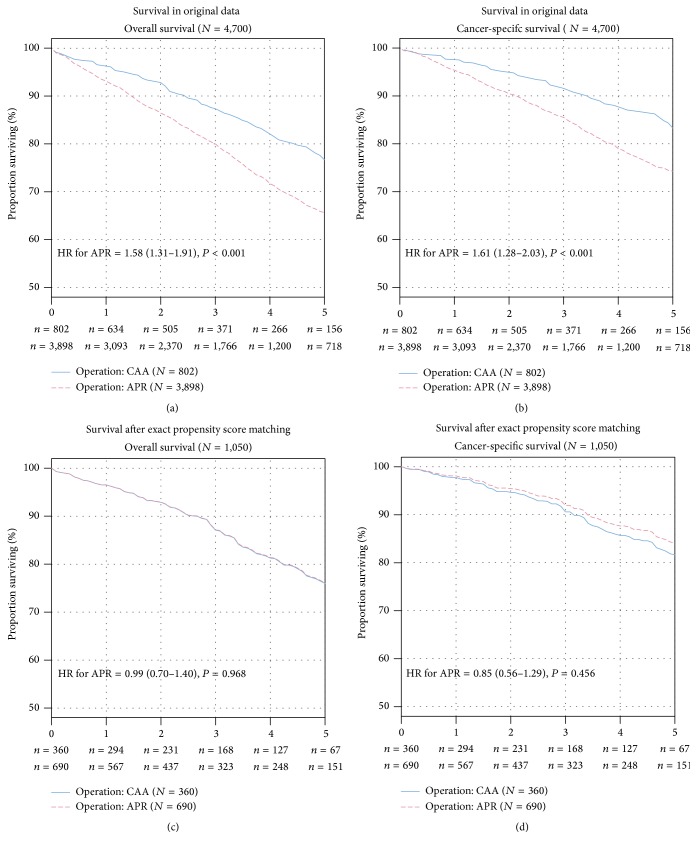
Kaplan-Meier curves for overall (panels a, c) and cancer-specific (panels b, d) survival in unadjusted and propensity score adjusted analysis. The overall survival (panels a, c) and cancer-specific survival (panels b, d) in unadjusted and propensity score adjusted analysis are depicted. The number of rectal cancer patients at risk in the two groups is given below each plot. HR: hazard ratio for APR compared with CAA with *P* value from likelihood-ratio test.

Panels (a) and (b) in Figure 2 display the Kaplan-Meier curves for overall and cancer-specific survival in patients with APR and CAA. In unadjusted Cox proportional hazards regression analysis, patients undergoing APR had a 58% increased risk of overall mortality (HR = 1.58, 95% CI: 1.31 to 1.91, *P* < 0.001) and a 61% increased risk of cancer-specific mortality (HR = 1.61, 95% CI: 1.28 to 2.03, *P* < 0.001). The 5-year overall survival for patients with APR was 65.6% (95% CI: 63.6 to 67.7%) compared with 76.7% (95% CI: 72.5 to 81.0%) for patients undergoing CAA (*P* < 0.001). The 5-year cancer-specific survival for patients with APR was 74.3% (95% CI: 72.4 to 76.2%) compared with 83.3% (95% CI: 79.5 to 87.3%) for patients undergoing CAA. After multivariable risk adjustment in the Cox regression analysis (Table 2), APR was persistently associated with an increased risk of overall mortality (hazard ratio of death = 1.37, 95% CI: 1.13 to 1.67, *P* = 0.001) and cancer-specific mortality (hazard ratio of death = 1.39, 95% CI: 1.10 to 1.76, *P* = 0.004). These results were additionally confirmed after variable selection (Table 2).

### 3.4. Adjusting for Patient Characteristics with Propensity Score Matching

To further corroborate the bias for APR in the patient characteristics and its potential influence on survival, logistic regression analysis with multivariable adjustment was performed (Table 3). Patients undergoing APR had more advanced cancer stages and more radiotherapy treatments, were significantly older, were less often African-Americans, and were less often married.

For exact propensity score matching, 3,650 patients were excluded because they did not have a counterpart in the other group who had exactly the same values for all baseline covariates. In the remaining 1,050 patients, no differences between patients undergoing APR and CAA were observed (for all covariates, *P* = 1.0), demonstrating a perfect matching. In Cox regression analyses after propensity score matching, the risk of overall mortality (HR of death = 0.99, 95% CI: 0.70 to 1.40, *P* = 0.968) and cancer-specific mortality (HR of death = 0.85, 95% CI: 0.56 to 1.29, *P* = 0.456) was not increased in patients undergoing APR. In the propensity score-matched analysis, the 5-year overall survival for patients undergoing APR was 76.1% (95% CI: 71.9 to 80.5%) compared with 76.0% (95% CI: 70.4 to 81.9%) for patients undergoing CAA (Figure 2). The 5-year cancer-specific survival in patients undergoing APR was 84.1% (95% CI: 80.5 to 88.0%) compared with 81.7% (95% CI: 76.5 to 87.2%) in patients undergoing CAA (Figure 2).

## 4. Discussion and Conclusions

The present study is, to the best of our knowledge, the first SEER analysis applying propensity score matching to determine the prognostic relevance of APR versus CAA. Based on the assessed cohort of nonmetastatic rectal cancer patients, the current study revealed the following two central results.

First, the rate of APR declined from 31.8% in 1998 to 19.2% in 2011, with a significant change in this trend at the end of 2004. Second, APR was associated with a significant survival disadvantage in univariate analysis and after conventional multivariable adjustment. This finding was in contrast to the lack of influence of APR on survival when optimally adjusting by exact propensity score matching. Consequently, the association between APR and worse survival observed in conventional analysis is not due to the APR itself but caused by highly biased patient characteristics.

The decline in the APR rate confirms previous research that has indicated a rate of 23% decrease in nonrestorative rectal resections between 2005 and 2010 in the regions covered by the SEER registry [15, 20]. In England, analysis of the national administrative database between 1996 and 2004 demonstrated that the APR rate significantly decreased from 29% to 21% [20]. Besides the declining trend of APR, the rates of APR vary immensely within the literature. In their retrospective analysis of discharge data from 21 states in the US from 2002 to 2004, Ricciardi et al. documented an APR rate of 50% [31].

The trend change in 2004 observed in the present investigation might be explained by the increasing implementation of preoperative chemoradiotherapy at that time. In 2004, Sauer et al. demonstrated better local control and a decreased rate of APR in patients with preoperative compared to postoperative chemoradiotherapy [32]. Another reason for the trend change in 2004 might be the increased use of phased array coil MRI [33], which proved to be a more accurate diagnostic technique in the prediction of a positive circumferential resection margin and sphincter infiltration and might thus have minimized potential overtreatment by APR. Furthermore, the more prevalent use of stapling devices might have contributed to the declining rate of APR [34, 35]. Inevitably, for a portion of patients, that is, those with sphincter-infiltrating tumors, APR is still the only curative treatment. In the future, the application of preoperative, targeted therapy to nonmetastatic rectal cancer could further reduce the nearly stagnating rate [36].

The risk of mortality for APR compared to CAA was exclusively analyzed in patients diagnosed after 2005, a time period with only moderate changes in APR rate. Thus, the time-trend bias was minimized. In this analysis, APR was associated with a significantly increased risk of mortality, which was approximately 60% after univariate analysis and approximately 38% after conventional multivariable adjustment. In contrast, after exact propensity score matching, no increased risk of mortality was observed after APR. To elaborate this discrepancy, patient and tumor characteristics were considered.

Of note, conventional multivariable analysis cannot fully adjust for confounders; for example, it cannot take into account the combined effect of two confounders (e.g., age and gender). Furthermore, effects such as collinearity cannot be ruled out. In contrast, the exact weighted propensity score matching that was applied in the present study is a modern, superior statistical method of building two identical groups, thus simulating randomization and precluding selection bias [28–30]. There was a statistically significant and clinically relevant bias in patient and tumor characteristics between the APR and CAA groups that strongly favored CAA against APR. Independent risk factors for a poor oncologic outcome after APR [5] occurred more often in the APR group. Patients undergoing APR were older, had more advanced cancer stages, and had fewer regional lymph nodes retrieved. The risk of mortality after APR versus CAA decreased with a higher degree of adjustment and was zero when an optimal adjustment was performed by exact propensity score matching. Hence, the association between APR and worse overall and cancer-specific survival is not caused by APR itself but rather reflects disadvantageous patient and tumor characteristics.

The relationship between the level of adjustment for patient and tumor characteristics and the oncologic outcome explains some of the contradictory findings in the literature [1, 3, 5, 6, 17–19, 37, 38]. A SEER-based analysis from 1998 to 2007 found a 35% increased risk of mortality for APR after conventional multivariable-adjusted Cox regression [37]. A Swedish population-based analysis from 1995 to 2003 [17] and two single center analyses from 1989 to 2002 and from 1990 to 2006 did not find such a negative impact [18, 19]. In contrast, in a pooled analysis of five European trials between 1987 and 2003 published by den Dulk et al., APR was associated with a higher rate of a positive circumferential margin and of local recurrences as well as decreased survival, although the likelihood of undergoing APR was included in the multivariable analysis [5]. Another investigation of the data in the Dutch Surgical Colorectal Audit conducted between 2010 and 2011 by the same research group did not find an increased rate of positive circumferential margin after APR [38].

The recent introduction of a more radical operative technique might explain potential improvement in oncological outcome after APR. In 2005, Marr and coauthors showed that in standard APR the specimen has a smaller diameter at the location of the tumor compared to anterior resection with total mesorectal excision (TME). The consequences of a smaller diameter were a smaller median distance from the tumor to the circumferential resection margin (CRM) and more CRM positive specimens [2]. At the beginning of the 21st century, Holm et al. started to perform more extensive APR, stopping the abdominal dissection above the beginning of the levators and dissecting more radically from beneath to completely remove these muscles [39]. West et al. demonstrated in 2010 that this cylindrical or extralevator APR approach led to reduced rates of positive CRM and intraoperative perforations [40]. Recent systematic reviews and meta-analyses showed significantly fewer local recurrences after the more extensive procedure [41, 42]. Data on the effect on survival is still scarce; some studies show an improvement [43], whereas smaller analyses have not yielded significantly better oncological outcomes compared with standard APR [34, 44]. The partial or complete adaption of this new form of APR in the last years, which is not referred to in most studies, might add to the contradictory results regarding oncologic outcome.

We would like to acknowledge the limitations of the present investigation. First, data corresponding to tumor height, adjuvant therapy, comorbidities, quality of TME, and CRM involvement are not available in the SEER registry. Therefore, the extent to which these parameters might have influenced prognosis remains unclear. Although we performed risk adjustment for known confounders, potential bias due to unknown confounding cannot be excluded. Additionally, survival is not the only oncological outcome in cancer patient care. Continence, genitourinary function and the superordinate criterion of quality of life are essential for deciding what type of operation to perform. Unfortunately, the SEER database does not provide data about quality of life. According to a recent Cochrane meta-analysis, reliable conclusions concerning quality of life after APR versus CAA have not been possible to date [45]. Additionally, data about postoperative morbidity after APR and CAA are sparse, with some evidence for a similar rate after both procedures [19].

The main strength of the present investigation comes from the great power associated with its large sample size. Because randomized controlled trials directly comparing results after APR and CAA are lacking and difficult to perform due to ethical reasons, the present analysis is probably the most appropriate study design.

## 5. Conclusion

In summary, the present population-based investigation on nonmetastatic rectal cancer patients provides evidence that APR itself is not associated with worse overall or cancer-specific survival. APR is performed in the presence of poor prognostic factors, such as age and tumor stage. Hence, overall and cancer-specific survival should not be an issue when deciding whether to perform APR.

## Figures and Tables

**Table 1:** Patient characteristics.

	Total	APR	CAA	*P* ^A^
	*N* = 4,700	*N* = 3,898	*N* = 802	
*Patient characteristics*				

Tumor stage (AJCC 6th ed.)				
Stage I	1106 (23.5%)	859 (22.0%)	247 (30.8%)	<0.001^A^
Stage IIA	1417 (30.1%)	1185 (30.4%)	232 (28.9%)	
Stage IIB	160 (3.4%)	150 (3.8%)	10 (1.2%)	
Stage IIIA	283 (6.0%)	219 (5.6%)	64 (8.0%)	
Stage IIIB	1159 (24.7%)	993 (25.5%)	166 (20.7%)	
Stage IIIC	575 (12.2%)	492 (12.6%)	83 (10.3%)	

Retrieved regional lymph nodes				
<12	2180 (46.4%)	1845 (47.3%)	335 (41.8%)	0.004^A^
12+	2520 (53.6%)	2053 (52.7%)	467 (58.2%)	

Grading				
G1	338 (7.2%)	264 (6.8%)	74 (9.2%)	0.007^A^
G2	3307 (70.4%)	2731 (70.1%)	576 (71.8%)	
G3/4	686 (14.6%)	581 (14.9%)	105 (13.1%)	
Unknown	369 (7.9%)	322 (8.3%)	47 (5.9%)	

Radiation				
None	1165 (24.8%)	897 (23.0%)	268 (33.4%)	<0.001^A^
Before surgery	2953 (62.8%)	2512 (64.4%)	441 (55.0%)	
After surgery	582 (12.4%)	489 (12.5%)	93 (11.6%)	

Year of diagnosis				
2005	744 (15.8%)	620 (15.9%)	124 (15.5%)	0.070^A^
2006	671 (14.3%)	566 (14.5%)	105 (13.1%)	
2007	688 (14.6%)	568 (14.6%)	120 (15.0%)	
2008	636 (13.5%)	547 (14.0%)	89 (11.1%)	
2009	653 (13.9%)	526 (13.5%)	127 (15.8%)	
2010	685 (14.6%)	572 (14.7%)	113 (14.1%)	
2011	623 (13.3%)	499 (12.8%)	124 (15.5%)	

Age				
<50	799 (17.0%)	629 (16.1%)	170 (21.2%)	<0.001^A^
50–64	1854 (39.4%)	1497 (38.4%)	357 (44.5%)	
65–79	1564 (33.3%)	1342 (34.4%)	222 (27.7%)	
80+	483 (10.3%)	430 (11.0%)	53 (6.6%)	

Gender				
Male	2972 (63.2%)	2477 (63.5%)	495 (61.7%)	0.329^A^
Female	1728 (36.8%)	1421 (36.5%)	307 (38.3%)	

Ethnicity				
Caucasian	3940 (83.8%)	3275 (84.0%)	665 (82.9%)	0.011^A^
African-American	366 (7.8%)	285 (7.3%)	81 (10.1%)	
Other/unknown	394 (8.4%)	338 (8.7%)	56 (7.0%)	
Marital status				
Married	2822 (60.0%)	2296 (58.9%)	526 (65.6%)	0.002^A^
Single/widowed	1174 (25.0%)	1003 (25.7%)	171 (21.3%)	
Other/unknown	704 (15.0%)	599 (15.4%)	105 (13.1%)	

*Outcome variables*				

Cause of death				
Alive	3668 (78.0%)	2988 (76.7%)	680 (84.8%)	<0.001^A^
Dead from cancer	714 (15.2%)	631 (16.2%)	83 (10.3%)	
Dead not from cancer	318 (6.8%)	279 (7.2%)	39 (4.9%)	

Follow-up				
Months	35.5 (23.4)	35.4 (23.4)	36.3 (23.8)	0.353^B^

*n* (%) and mean (SD).

^A^Chi-square test ^B^Mann–Whitney *U* test.

**Table 2:** Prognostic factors for overall and cancer-specific mortality.

	Overall mortality						Cancer-specific mortality					
	Unadjusted^A^		Cox regression, full model^B^		Cox regression, variable selection^C^		Unadjusted^A^		Cox regression, full model^B^		Cox regression, variable selection^C^	
	HR (95% CI)	*P* ^D^	HR (95% CI)	*P* ^D^	HR (95% CI)	*P* ^D^	HR (95% CI)	*P* ^D^	HR (95% CI)	*P* ^D^	HR (95% CI)	*P* ^D^
Operation												
CAA	Reference	<0.001	Reference	0.001	Reference	0.001	Reference	<0.001	Reference	0.004	Reference	0.004
APR	1.58 (1.31–1.91)		1.37 (1.13–1.67)		1.36 (1.12–1.64)		1.61 (1.28–2.03)		1.39 (1.10–1.76)		1.39 (1.11–1.76)	

Tumor stage (AJCC 6th ed.)												
Stage I	Reference	<0.001	Reference	<0.001	Reference	<0.001	Reference	<0.001	Reference	<0.001	Reference	<0.001
Stage IIA	1.23 (1.02–1.50)		1.47 (1.20–1.80)		1.49 (1.22–1.82)		1.67 (1.28–2.18)		1.90 (1.44–2.50)		1.91 (1.45–2.51)	
Stage IIB	2.17 (1.58–2.99)		2.71 (1.96–3.76)		2.67 (1.93–3.70)		3.83 (2.63–5.59)		4.51 (3.06–6.65)		4.45 (3.02–6.55)	
Stage IIIA	1.50 (1.11–2.01)		1.82 (1.34–2.46)		1.82 (1.34–2.46)		2.13 (1.45–3.11)		2.50 (1.69–3.69)		2.47 (1.67–3.64)	
Stage IIIB	1.76 (1.46–2.13)		2.28 (1.87–2.80)		2.29 (1.87–2.81)		2.81 (2.18–3.63)		3.39 (2.59–4.43)		3.38 (2.58–4.42)	
Stage IIIC	3.42 (2.81–4.17)		4.11 (3.33–5.08)		4.09 (3.31–5.06)		5.90 (4.56–7.65)		6.57 (4.99–8.65)		6.54 (4.97–8.61)	

Retrieved regional lymph nodes												
<12	Reference	0.391	Reference	<0.001	Reference	<0.001	Reference	0.576	Reference	0.001	Reference	0.001
12+	0.95 (0.84–1.07)		0.79 (0.69–0.90)		0.78 (0.69–0.88)		0.96 (0.83–1.11)		0.77 (0.66–0.90)		0.76 (0.65–0.89)	

Grading												
G1	Reference	<0.001	Reference	0.006	Reference	0.004	Reference	<0.001	Reference	<0.001	Reference	<0.001
G2	1.43 (1.08–1.89)		1.26 (0.95–1.68)		1.28 (0.96–1.70)		2.29 (1.50–3.52)		1.92 (1.25–2.95)		1.95 (1.27–3.00)	
G3/4	2.15 (1.59–2.92)		1.59 (1.17–2.16)		1.61 (1.19–2.19)		4.15 (2.66–6.46)		2.80 (1.79–4.38)		2.84 (1.82–4.45)	
Unknown	1.07 (0.74–1.55)		1.15 (0.79–1.66)		1.16 (0.80–1.68)		1.63 (0.97–2.75)		1.55 (0.92–2.61)		1.59 (0.94–2.68)	

Radiation												
None	Reference	<0.001	Reference	<0.001	Reference	<0.001	Reference	<0.001	Reference	<0.001	Reference	<0.001
Before surgery	0.58 (0.51–0.67)		0.56 (0.48–0.66)		0.56 (0.48–0.66)		0.74 (0.62–0.87)		0.62 (0.51–0.75)		0.61 (0.51–0.74)	
After surgery	0.82 (0.68–0.99)		0.62 (0.51–0.76)		0.63 (0.51–0.77)		1.00 (0.80–1.25)		0.63 (0.49–0.80)		0.64 (0.50–0.81)	

Year												
2005	Reference	0.068	Reference	0.141	—	—	Reference	0.029	Reference	0.072	—	—
2006	0.94 (0.79–1.12)		0.99 (0.83–1.18)		—	—	0.97 (0.79–1.20)		1.02 (0.83–1.25)		—	—
2007	0.85 (0.71–1.03)		0.85 (0.71–1.03)		—	—	0.86 (0.69–1.08)		0.86 (0.69–1.09)		—	—
2008	0.95 (0.77–1.17)		0.99 (0.80–1.22)		—	—	1.00 (0.78–1.28)		1.02 (0.80–1.31)		—	—
2009	0.88 (0.69–1.12)		1.06 (0.83–1.35)		—	—	0.83 (0.62–1.11)		0.96 (0.71–1.30)		—	—
2010	0.76 (0.56–1.03)		0.84 (0.62–1.15)		—	—	0.60 (0.40–0.90)		0.65 (0.44–0.98)		—	—
2011	0.43 (0.22–0.82)		0.51 (0.26–0.97)		—	—	0.36 (0.15–0.89)		0.40 (0.16–0.99)		—	—

Age												
<50	Reference	<0.001	Reference	<0.001	Reference	<0.001	Reference	<0.001	Reference	<0.001	Reference	<0.001
50–64	1.07 (0.86–1.32)		1.10 (0.89–1.37)		1.09 (0.88–1.35)		0.96 (0.76–1.21)		1.01 (0.80–1.27)		1.00 (0.79–1.27)	
65–79	1.80 (1.47–2.21)		1.76 (1.43–2.16)		1.74 (1.42–2.15)		1.37 (1.09–1.72)		1.37 (1.09–1.73)		1.38 (1.09–1.74)	
80+	3.88 (3.10–4.86)		3.25 (2.56–4.12)		3.14 (2.47–3.98)		2.49 (1.92–3.25)		2.23 (1.68–2.96)		2.21 (1.67–2.93)	

Gender												
Male	Reference	0.012	Reference	0.282	—	—	Reference	0.066	Reference	0.641	—	—
Female	1.18 (1.04–1.33)		0.93 (0.81–1.06)		—	—	1.15 (0.99–1.34)		0.96 (0.82–1.13)		—	—

Ethnicity												
Caucasian	Reference	0.122	Reference	0.152	—	—	Reference	0.061	Reference	0.145	Reference	0.135
African-American	1.24 (1.00–1.54)		1.25 (1.00–1.55)		—	—	1.34 (1.04–1.72)		1.30 (1.00–1.67)		1.30 (1.01–1.68)	
Other/unknown	0.95 (0.75–1.19)		1.02 (0.80–1.28)		—	—	0.91 (0.68–1.21)		0.97 (0.73–1.30)		0.96 (0.72–1.28)	

Marital status												
Married	Reference	<0.001	Reference	<0.001	Reference	<0.001	Reference	<0.001	Reference	0.001	Reference	0.001
Single/widowed	1.81 (1.57–2.07)		1.42 (1.22–1.65)		1.41 (1.22–1.62)		1.66 (1.41–1.97)		1.34 (1.12–1.60)		1.31 (1.10–1.56)	
Other/unknown	1.41 (1.19–1.67)		1.41 (1.18–1.68)		1.41 (1.19–1.68)		1.44 (1.17–1.76)		1.37 (1.11–1.69)		1.35 (1.10–1.66)	

Hazard ratios (HR) with 95% confidence intervals; ^A^univariate Cox regression analysis; ^B^multivariable Cox regression analysis full model; ^C^backward variable selection from full model multivariable Cox regression analysis full model; ^D^likelihood ratio tests.

**Table 3:** Bias for abdominoperineal resection.

	Logistic regression in raw data (*N* = 4,700)^A^		Patient characteristics after exact propensity score matching (*N* = 1,050)^C^		
	OR (95% CI)	*P* ^B^	Total *N* = 1,050	APR *N* = 690	CAA *N* = 360
Tumor stage (AJCC 6th ed.)					
Stage I	Reference	<0.001	295.2 (28.1%)	194 (28.1%)	101.2 (28.1%)
Stage IIA	1.21 (0.98–1.51)		398.7 (38.0%)	262 (38.0%)	136.7 (38.0%)
Stage IIB	3.53 (1.91–7.31)		1.5 (0.1%)	1 (0.1%)	0.5 (0.1%)
Stage IIIA	0.90 (0.65–1.25)		36.5 (3.5%)	24 (3.5%)	12.5 (3.5%)
Stage IIIB	1.42 (1.12–1.80)		261.7 (24.9%)	172 (24.9%)	89.7 (24.9%)
Stage IIIC	1.52 (1.14–2.04)		56.3 (5.4%)	37 (5.4%)	19.3 (5.4%)

Retrieved regional lymph nodes					
<12	Reference	0.155	503.7 (48.0%)	331 (48.0%)	172.7 (48.0%)
12+	0.89 (0.75–1.05)		546.3 (52.0%)	359 (52.0%)	187.3 (52.0%)

Grading					
G1	Reference	0.072	22.8 (2.2%)	15 (2.2%)	7.8 (2.2%)
G2	1.22 (0.92–1.61)		969.3 (92.3%)	637 (92.3%)	332.3 (92.3%)
G3/4	1.34 (0.95–1.88)		41.1 (3.9%)	27 (3.9%)	14.1 (3.9%)
Unknown	1.69 (1.12–2.57)		16.7 (1.6%)	11 (1.6%)	5.7 (1.6%)

Radiation					
None	Reference	<0.001	228.3 (21.7%)	150 (21.7%)	78.3 (21.7%)
Before surgery	1.75 (1.43–2.13)		768.5 (73.2%)	505 (73.2%)	263.5 (73.2%)
After surgery	1.58 (1.20–2.10)		53.3 (5.1%)	35 (5.1%)	18.3 (5.1%)

Year					
2005	Reference	0.155	176.5 (16.8%)	116 (16.8%)	60.5 (16.8%)
2006	1.05 (0.79–1.41)		152.2 (14.5%)	100 (14.5%)	52.2 (14.5%)
2007	0.92 (0.69–1.23)		149.1 (14.2%)	98 (14.2%)	51.1 (14.2%)
2008	1.19 (0.88–1.62)		94.3 (9.0%)	62 (9.0%)	32.3 (9.0%)
2009	0.82 (0.62–1.09)		172 (16.4%)	113 (16.4%)	59 (16.4%)
2010	0.98 (0.73–1.31)		172 (16.4%)	113 (16.4%)	59 (16.4%)
2011	0.81 (0.61–1.08)		133.9 (12.8%)	88 (12.8%)	45.9 (12.8%)

Age					
<50	Reference	<0.001	132.4 (12.6%)	87 (12.6%)	45.4 (12.6%)
50–64	1.20 (0.97–1.48)		544.8 (51.9%)	358 (51.9%)	186.8 (51.9%)
65–79	1.86 (1.48–2.33)		343.9 (32.8%)	226 (32.8%)	117.9 (32.8%)
80+	2.76 (1.96–3.94)		28.9 (2.8%)	19 (2.8%)	9.9 (2.8%)

Gender					
Male	Reference	0.066	833.9 (79.4%)	548 (79.4%)	285.9 (79.4%)
Female	0.86 (0.73–1.01)		216.1 (20.6%)	142 (20.6%)	74.1 (20.6%)

Ethnicity					
Caucasian	Reference	0.008	1033.3 (98.4%)	679 (98.4%)	354.3 (98.4%)
African-American	0.71 (0.55–0.94)		7.6 (0.7%)	5 (0.7%)	2.6 (0.7%)
Other/unknown	1.30 (0.97–1.77)		9.1 (0.9%)	6 (0.9%)	3.1 (0.9%)

Marital status					
Married	Reference	0.006	897.8 (85.5%)	590 (85.5%)	307.8 (85.5%)
Single/widowed	1.29 (1.06–1.58)		106.5 (10.1%)	70 (10.1%)	36.5 (10.1%)
Other/unknown	1.33 (1.06–1.69)		45.7 (4.3%)	30 (4.3%)	15.7 (4.3%)

After exclusion of 3,650 patients for the exact propensity score matching, no bias was observed in the remaining 1,050 patients for APR versus CAA (all *P* = 1).

^A^Multivariable logistic regression with the odds ratio (OR) for APR in the original raw data set (*N* = 4,700).

^B^Likelihood ratio tests.

^C^All  *P* = 1 for comparison of APR versus CAA in weighted Chi-square tests after exact weighted propensity score matching (*N* = 1,050).

Weighted matching causes decimals for the number of patients in the group with CAA.
